# Correction: Pancreatic cancer-initiating cell exosome message transfer into noncancer-initiating cells: the importance of CD44v6 in reprogramming

**DOI:** 10.1186/s13046-024-03216-3

**Published:** 2024-10-31

**Authors:** Zhe Wang, Hanxue Sun, Jan Provaznik, Thilo Hackert, Margot Zöller

**Affiliations:** 1 Department of Oncology, The First Affiliated Hospital of Guangdong Pharmaceutical University, Guangzhou, China; 2 Pancreas Section, University Hospital of Surgery, Im Neuenheimer Feld 110, D69120 Heidelberg, Germany; 3 Gene Core Unit, EMBL, Heidelberg, Germany

**Correction: J Exp Clin Cancer Res 38, 132 (2019)**

10.1186/s13046-019-1129-8

Following publication of the original article [1], the authors identified errors in the figures, specifically:

- Figure 3i - A818v6kd + CIC-TEX VEGRF3
- Figure 4g - A818 Casp9 and A818 v6kd MDR1
- Figure 7f - A818 neg control and A818 + GEM CD44v6

The corrections do not have any effect on the results or conclusions of the paper. The correct figures are presented below:

**Incorrect Fig.** **3****i**

**Fig. 3:**
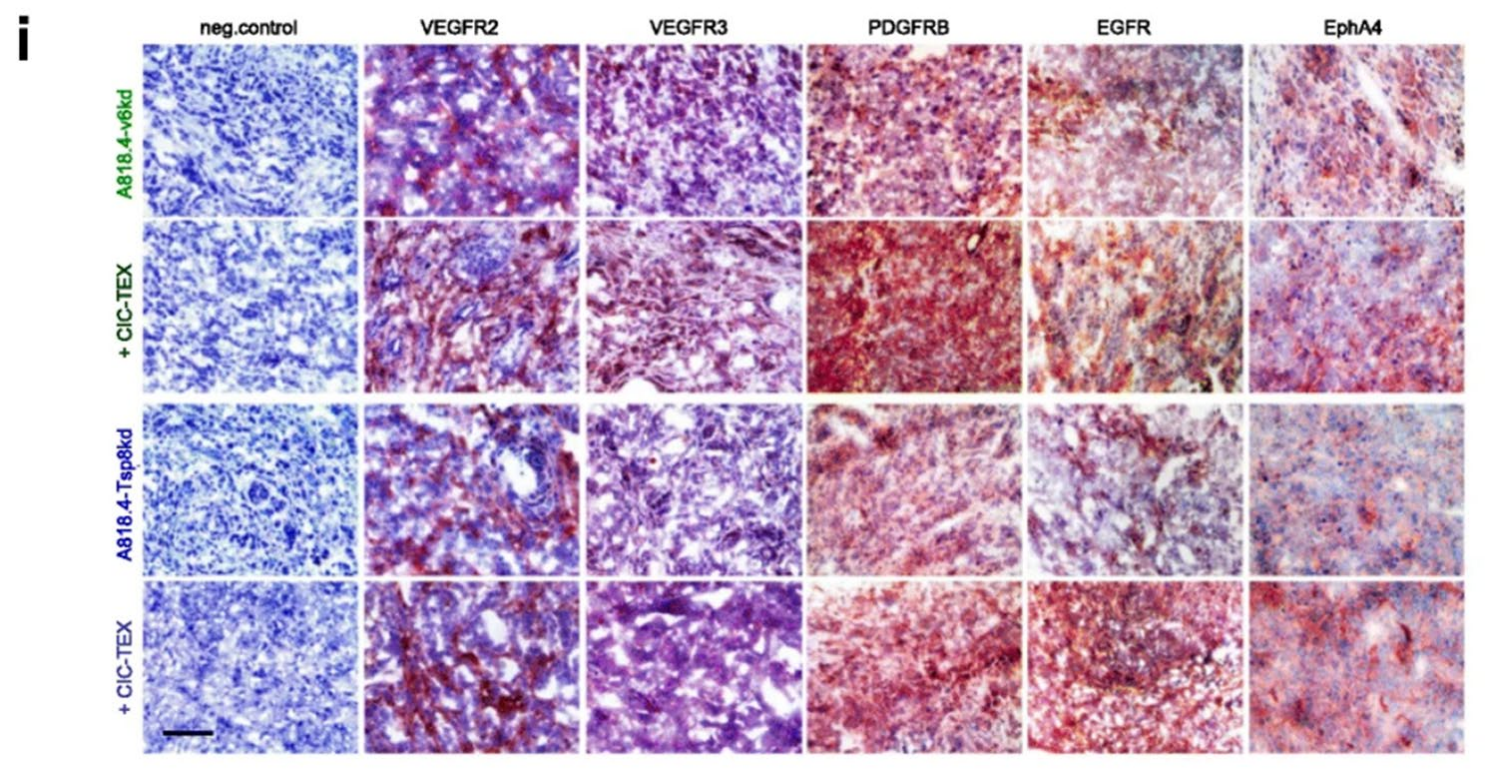
CIC-TEX-initiated changes in RTK and downstream signaling molecules in CD44v6kd and Tspan8kd cells. **a** Signaling array of A818.4-v6kd cells cultured for 72 h with/without CIC-TEX. The relative signal strength was evaluated by ImageJ; significant differences by coculture with CICTEX: *. Flow-cytometry and WB analysis of (**b**,** c**) RTK expression in kd-TEX- or CIC-TEX-treated kd cells and (**d**,** e**) major pathway-engaged cytosolic signaling molecules; b,d mean % stained cells ± SD (3 assays), significant differences by coculture with TEX: *; **c**,** e** representative examples and relative signal strength ± SD of 3 independent experiments including p-values for kd cells compared to CIC-TEX-treated kd cells; **f** pathways from miRNA to RTK (IPA-based STRING analysis after predicted target mRNA selection by microrna.org and targetscan.org) for ≥ 2-fold upregulated miRNA (framed) and ≥ 2-fold reduced mRNA recovery in CIC-TEX-treated v6kd or Tsp8kd cells compared to untreated kd cells; **g** IPA-based STRING analysis after predicted target mRNA selection by microrna.org and targetscan.org for ≥ 2-fold reduced miRNA in CIC-TEX-treated compared to untreated v6kd cells and of ≥ 2-fold upregulated predicted mRNA targets that are engaged in signal transduction. **h** Flow-cytometry analysis of ex vivo harvested intrapancreatic A818.4-v6kd cells from nude mice with/without weekly iv CIC-TEX treatment; mean % stained cells ± SD (3 tumors), significant differences by CIC-TEX treatment: s; **i** Representative immunohistology examples of A818.4-v6kd and -Tsp8kd shockfrozen tumor sections with/without CIC-TEX treatment stained with the indicated antibodies (scale bar: 100 μm). (List of synonyms: Additional file 1: Table S1). CIC-TEX treatment strongly affects RTK expression and downstream signaling molecules in vitro and in vivo. Changes in the recovery of mRNA engaged in signal transduction (Additional file 1: Figure S2d, S2e) are accompanied at a noteworthy frequency by reversely altered miRNA expression in CIC-TEX-treated kd, predominantly v6kd cells

**Correct Fig.** **3****i**

**Fig. 3:**
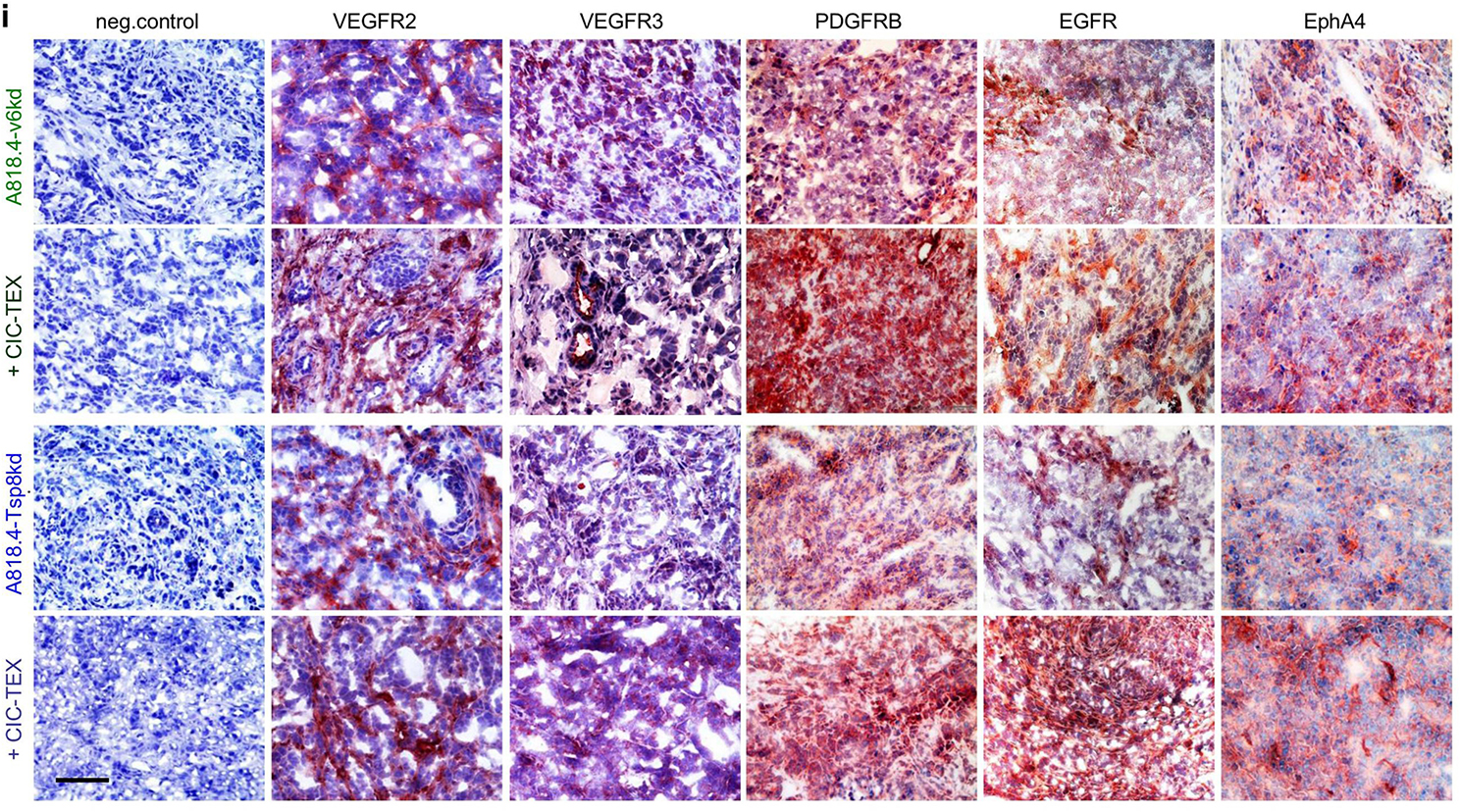
CIC-TEX-initiated changes in RTK and downstream signaling molecules in CD44v6kd and Tspan8kd cells. **a** Signaling array of A818.4-v6kd cells cultured for 72 h with/without CIC-TEX. The relative signal strength was evaluated by ImageJ; significant differences by coculture with CICTEX: *. Flow-cytometry and WB analysis of (**b**, **c**) RTK expression in kd-TEX- or CIC-TEX-treated kd cells and (**d**,** e**) major pathway-engaged cytosolic signaling molecules; b,d mean % stained cells ± SD (3 assays), significant differences by coculture with TEX: *; **c**,** e** representative examples and relative signal strength ± SD of 3 independent experiments including p-values for kd cells compared to CIC-TEX-treated kd cells; **f** pathways from miRNA to RTK (IPA-based STRING analysis after predicted target mRNA selection by microrna.org and targetscan.org) for ≥ 2-fold upregulated miRNA (framed) and ≥ 2-fold reduced mRNA recovery in CIC-TEX-treated v6kd or Tsp8kd cells compared to untreated kd cells; **g** IPA-based STRING analysis after predicted target mRNA selection by microrna.org and targetscan.org for ≥ 2-fold reduced miRNA in CIC-TEX-treated compared to untreated v6kd cells and of ≥ 2-fold upregulated predicted mRNA targets that are engaged in signal transduction. **h** Flow-cytometry analysis of ex vivo harvested intrapancreatic A818.4-v6kd cells from nude mice with/without weekly iv CIC-TEX treatment; mean % stained cells ± SD (3 tumors), significant differences by CIC-TEX treatment: s; **i** Representative immunohistology examples of A818.4-v6kd and -Tsp8kd shockfrozen tumor sections with/without CIC-TEX treatment stained with the indicated antibodies (scale bar: 100 μm). (List of synonyms: Additional file 1: Table S1). CIC-TEX treatment strongly affects RTK expression and downstream signaling molecules in vitro and in vivo. Changes in the recovery of mRNA engaged in signal transduction (Additional file 1: Figure S2d, S2e) are accompanied at a noteworthy frequency by reversely altered miRNA expression in CIC-TEX-treated kd, predominantly v6kd cells

**Incorrect Fig.** **4****g**

**Fig. 4:**
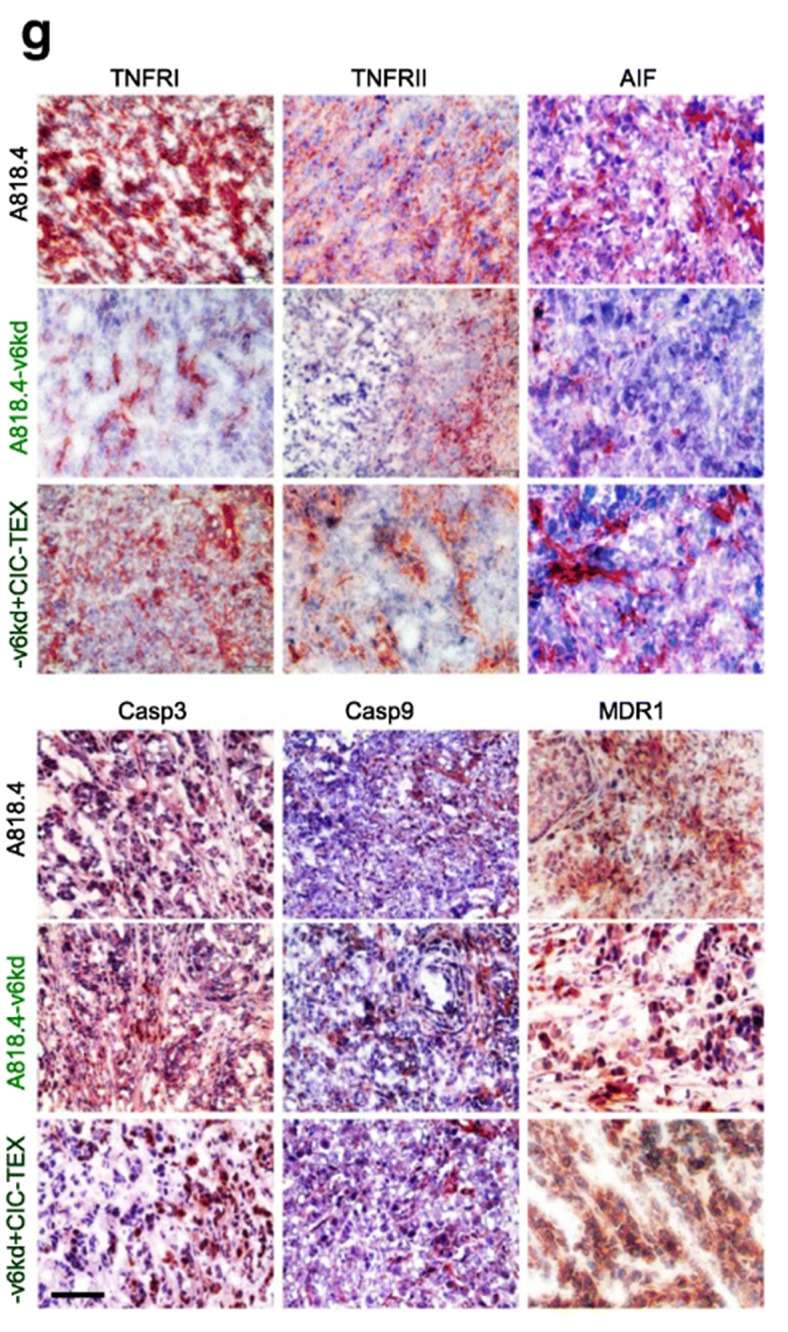
CIC-TEX-promoted apoptosis-resistance in CD44v6kd and/or Tspan8kd cells. **a** Flow-cytometry of cells cultured for 48 h in the presence of cisplatin; mean % AnnexinV + and AnnexinV+/PI + cells ± SD (triplicates); significant differences of wt cells versus CIC or kd cells: *; significant differences by CIC-TEX treatment: s. **b** mRNA that expression differed by ≥ 2-fold after CIC-TEX treatment in Tsp8kd (blue) or v6kd (violet) cells were sorted by KEGG analysis according to the engagement in distinct apoptotic processes. **c-f** Flow-cytometry analysis of apoptosis-related receptor and cytoplasmic signaling molecules in A818.4, −v6kd and CIC-enriched cells and in v6kd cells cocultured with v6kd- or CIC-TEX; mean % stained cells ± SD (3 assays), significant differences between wt cells, v6kd cells and CIC: *, significant differences by coculture of v6kd cells with TEX: s. **g** Representative immunohistology examples of A818.4 and -v6kd shock-frozen tumor sections from untreated or CIC-TEX-treated mice stained with the indicated antibodies (scale bar: 100 μm). (List of synonyms: Additional file 1: Table S1). Slightly reduced apoptosis resistance of v6kd and Tsp8kd tumor cells becomes mitigated by CIC-TEX treatment. The impact of CIC-TEX on apoptosis resistance is mostly restricted to the regulation of molecules engaged in the intrinsic pathway of apoptosis induction. An exception is the increased expression of drug transporters in CIC-TEX-treated v6kd cells

**Correct Fig.** **4****g**

**Fig. 4:**
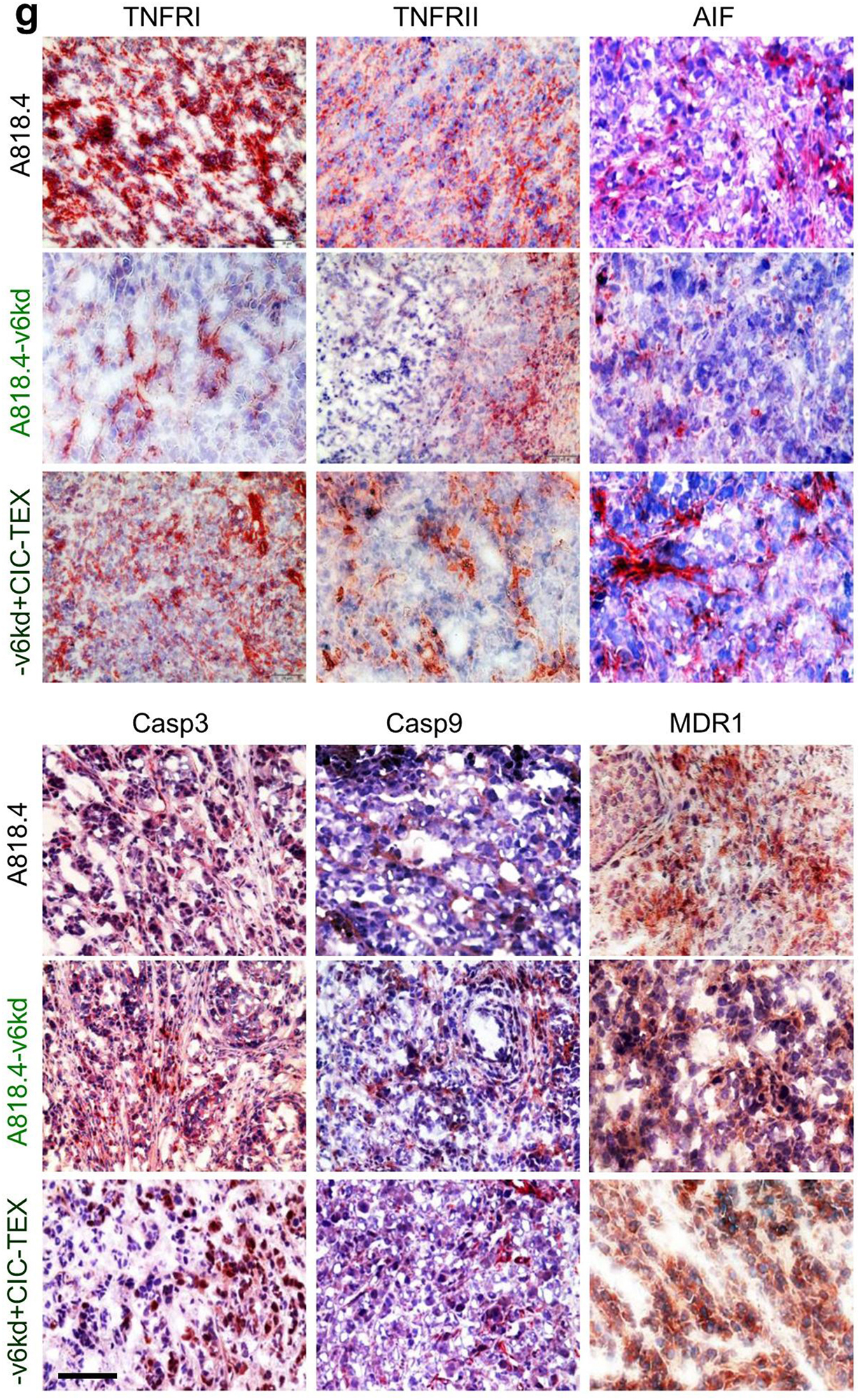
CIC-TEX-promoted apoptosis-resistance in CD44v6kd and/or Tspan8kd cells. **a** Flow-cytometry of cells cultured for 48 h in the presence of cisplatin; mean % AnnexinV + and AnnexinV+/PI + cells ± SD (triplicates); significant differences of wt cells versus CIC or kd cells: *; significant differences by CIC-TEX treatment: s. **b** mRNA that expression differed by ≥ 2-fold after CIC-TEX treatment in Tsp8kd (blue) or v6kd (violet) cells were sorted by KEGG analysis according to the engagement in distinct apoptotic processes. **c-f** Flow-cytometry analysis of apoptosis-related receptor and cytoplasmic signaling molecules in A818.4, −v6kd and CIC-enriched cells and in v6kd cells cocultured with v6kd- or CIC-TEX; mean % stained cells ± SD (3 assays), significant differences between wt cells, v6kd cells and CIC: *, significant differences by coculture of v6kd cells with TEX: s. **g** Representative immunohistology examples of A818.4 and -v6kd shock-frozen tumor sections from untreated or CIC-TEX-treated mice stained with the indicated antibodies (scale bar: 100 μm). (List of synonyms: Additional file 1: Table S1). Slightly reduced apoptosis resistance of v6kd and Tsp8kd tumor cells becomes mitigated by CIC-TEX treatment. The impact of CIC-TEX on apoptosis resistance is mostly restricted to the regulation of molecules engaged in the intrinsic pathway of apoptosis induction. An exception is the increased expression of drug transporters in CIC-TEX-treated v6kd cells

**Incorrect Fig.** **7****f**

**Fig. 7:**
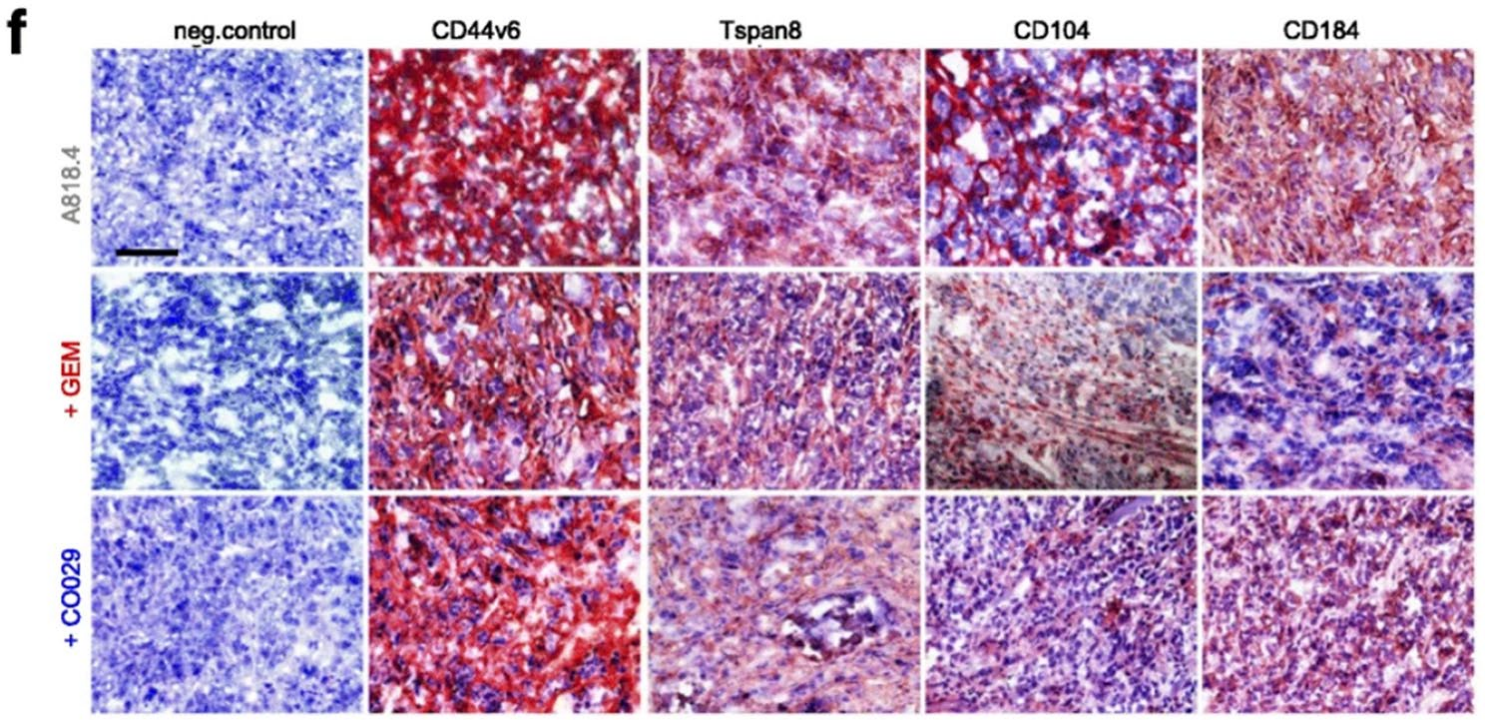
Binding and RTK inhibition for blocking CIC-TEX activity as adjuvant PaCa therapy. Mice received an ot (A818.4-v6kd) or a sc (A818.4) tumor cell injection and weekly the RTK inhibitor GEM (iv) or anti-Tsp8 (CO029) (iv). **a** Survival time and mean survival time of v6kd-TB mice (intrapancreatic) treated with CIC-TEX (2x/wk) and GEM or CO029 (1x/wk). **b** Recovery of disseminated tumor cells in cultures of dispersed organs at autopsy. **c** Tumor growth rate, survival time and mean survival time of sc A818.4-TB mice, treated with GEM or CO029; **d** disseminated tumor cell recovery in cultures of dispersed organs at autopsy; **a-d** p-values for the mean survival time and the numbers of organs containing dispersed tumor cells (after Bonferroni-Holm correction) of GEM- or CO029-treated compared to untreated v6kd-TB and CIC-TEX-treated-TB or wt-TB are indicated. **e**,** g**,**i**,** k**,**l** Flow-cytometry of dispersed tumor cells and BMC of untreated and GEM- or CO029-treated A818.4-TB mice evaluating CIC, angiogenic, apoptosis, proteolysis, adhesion (only tumor cells) marker expression and MDSC (CD11b + Gr1+) (only BMC); mean % stained cells ± SD (3 mice), significant differences by GEM- or CO029-treatment: *. **f**,** h**,**j** Immunohistology of A818.4 shock-frozen tumor sections from untreated, GEM- or CO029-treated mice stained for CIC markers, VEGFR2, VEGFR3, CD31, MMP2 and TIMP1 (scale bar: 100 μm). GEM and CO029 weakly affect tumor growth and strongly tumor cell dissemination. Ex vivo analysis indicates that GEM and CO029 act independently. GEM primarily affects tumor cell apoptosis, proteases and MDSC expansion, CO029 treatment is accompanied by a reduction in (Tsp8-associated) CIC markers and compromises (lymph)angiogenesis

**Correct Fig.** **7****f**

**Fig. 7:**
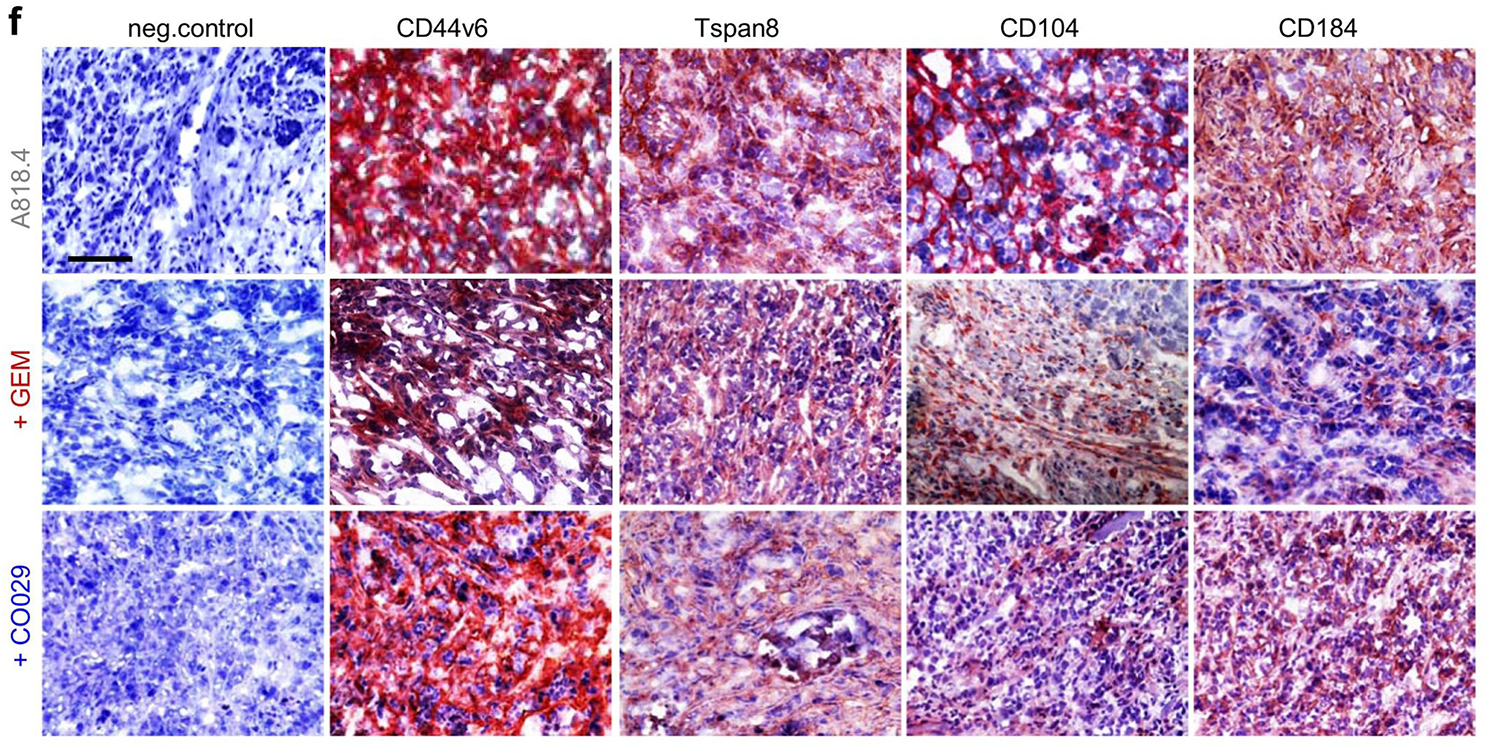
Binding and RTK inhibition for blocking CIC-TEX activity as adjuvant PaCa therapy. Mice received an ot (A818.4-v6kd) or a sc (A818.4) tumor cell injection and weekly the RTK inhibitor GEM (iv) or anti-Tsp8 (CO029) (iv). **a** Survival time and mean survival time of v6kd-TB mice (intrapancreatic) treated with CIC-TEX (2x/wk) and GEM or CO029 (1x/wk). **b** Recovery of disseminated tumor cells in cultures of dispersed organs at autopsy. **c** Tumor growth rate, survival time and mean survival time of sc A818.4-TB mice, treated with GEM or CO029; **d** disseminated tumor cell recovery in cultures of dispersed organs at autopsy; **a-d** p-values for the mean survival time and the numbers of organs containing dispersed tumor cells (after Bonferroni-Holm correction) of GEM- or CO029-treated compared to untreated v6kd-TB and CIC-TEX-treated-TB or wt-TB are indicated. **e**,** g**,**i**,** k**,**l** Flow-cytometry of dispersed tumor cells and BMC of untreated and GEM- or CO029-treated A818.4-TB mice evaluating CIC, angiogenic, apoptosis, proteolysis, adhesion (only tumor cells) marker expression and MDSC (CD11b + Gr1+) (only BMC); mean % stained cells ± SD (3 mice), significant differences by GEM- or CO029-treatment: *. **f**,** h**,**j** Immunohistology of A818.4 shock-frozen tumor sections from untreated, GEM- or CO029-treated mice stained for CIC markers, VEGFR2, VEGFR3, CD31, MMP2 and TIMP1 (scale bar: 100 μm). GEM and CO029 weakly affect tumor growth and strongly tumor cell dissemination. Ex vivo analysis indicates that GEM and CO029 act independently. GEM primarily affects tumor cell apoptosis, proteases and MDSC expansion, CO029 treatment is accompanied by a reduction in (Tsp8-associated) CIC markers and compromises (lymph)angiogenesis
